# Evaluate clinical outcomes of three single implants with non-splinted crowns and two implants supporting a three unit bridge

**DOI:** 10.6026/973206300220993

**Published:** 2026-02-28

**Authors:** Amit Gaurav, Rakhi Kumari, Ankur Joshi, Rahul Dami, Suruchi Singh, Sadaf Kazmi

**Affiliations:** 1 Department of Prosthodontics, Crown and Bridge Dentistry, Mithila Minority Dental College & Hospital, Darbhanga, India; 2 Department of Dentistry, Government Medical College and Hospital, Purnea, India; 3 Department of Dentistry, Government Medical College, Dehradun, India; 4 Department of Prosthodontics, Crown and Bridge Dentistry, Seema Dental College and Hospital Rishikesh, India; 5 Department of Prosthodontics and Crown & Bridge, Buddha Institute of Dental Sciences and Hospital, Patna, India

**Keywords:** Single implant, non-splinted crown, fixed dental prosthesis

## Abstract

There is still a dearth of literature comparing the use of two or three implants in the rehabilitation of three-unit edentulous regions in the posterior jaw region. Therefore, it is of interest to evaluate the clinical outcomes and complications of three single implants with non-splinted crowns (NSC) and two implants supporting a three unit bridge (FDP) with an intervening pontic. Details of 478 patients who had NSC and 456 patients who had FDP were obtained once they fulfilled the standards for inclusion as well as standards of exclusion. Mesial and distal bone height, mobility of implant, peri implantitis (PI) and loss of implants were among the biological complications that were evaluated. The crestal bone loss was lesser in three implants with non-splinted crowns than two implants supporting a three unit bridge with an intervening pontic. The overall complications were also greater in two implants supporting a three unit bridge as compared to three implants with non-splinted crowns.

## Background:

Regarding both the mandible and maxilla, dental implants have been essential to the therapy of patients who are partially edentulous [1,2-3]. Long-term achievement remains well documented. However, factors that help doctors choose the best prosthodontic and surgical strategy also have an impact on the final outcome. Selecting a prosthodontic portion made of splinted or non-splinted crowns is one of these criteria [4,5-6]. By distributing the occlusal forces applied to the implants, splinted crowns can lessen the stress on the adjacent peri-implant bone. It also reduces the likelihood of prosthodontic problems. Ethical restrictions prohibit the application of occlusal overloading in human subjects. Therefore, the research supporting this treatment approach is frequently carried out by finite and photoelastic assessments [7,8-9]. In a three- unit splinted crown restoration, a study showed a decreased total peak stress generation across the central implant. In contrast, in an unsplinted restoration, the stresses were focused around each of the loaded implants [6]. Another study reported similar findings, showing that splinted implant restorations placed less strain on the margin of crowns than non-splinted ones [2].

While there are benefits to splinted restorations, it is crucial to practice proper dental hygiene in the interproximal regions in order to prevent the occurrence of peri-implantitis (PI) [10,11- 12]. This would make 3 non-splinted crowns a better prosthodontic alternative than splinted crowns, especially for patients with poor man euverability when cleaning or previous episodes of periodontitis [13, 14-15]. Splinting implant-supported crowns has the added drawback of being difficult to fit the framework and provide a suitable emergence profile. The amount of implants needed to restore a partially dentulous area is another important consideration [16, 17,- 18]. This poses a dilemma to choose between an implant-retained bridge and one implant for each lost tooth. One implant per missing tooth appears to be a reasonable clinical option for lowering certain risk factors, like overload [19,20-21]. Nonetheless, a number of studies have shown that full-arch rehabilitation using cross-arch splinted prostheses, which are supported by fewer implants than missing teeth, can be accomplished successfully [21, 22- 23]. The application of three implants in the restoration of a three-unit edentulous area may be hampered by low bone quality and space constraints. By employing two implants that support a three unit bridge with an intervening pontic, this restriction can be removed [24, 25-26]. Moreover, a frequently disregarded determining aspect is the price. The clinical decision regarding therapy may be influenced by the choice between two and three implants [25, 26-27]. Comparing the entire cumulative cost of each therapy option-including any potential side effects-is crucial, though [28- 29]. There is still a dearth of literature comparing the use of two or three implants in the rehabilitation of three-unit edentulous regions in the posterior part of mandible as well as maxilla. Therefore, it is of interest to retrospectively evaluate the clinical outcomes and complications of three implants with non-splinted crowns (NSC) and two implants supporting a three unit bridge with an intervening pontic (FDP) in the rehabilitation of three-unit edentulous regions in the posterior part of mandible as well as maxilla.

## Methods and Materials:

The investigation was a descriptive retrospective analysis of implant cases. All successive instances of a NSC or FDP inserted in the back area of jaws during 6 years from May 2018 to May 2024 were taken into consideration. Applying the D4W dental software for practice administration (Dental4Windows, Centaur Ltd, Australia), information was gathered from patients' electronic records. The demographic information on the patients, which was anonymized, included their gender, years of age, history of illness, smoking status and the total amount of teeth they still had, excluding their third molars.

## Inclusion criteria and exclusion criteria:

All implant prostheses that were operational for a minimum of one year were included in the study. Cantilevered implants, implants supporting complete or removal partial dentures and any implant site that received bone graft material or a sinus lift procedure were excluded. Patients on bisphosphonate therapy or who had bone disease were excluded but patients with any other medical condition were included. Details of 478 patients who had NSC and 456 patients who had FDP were obtained once they fulfilled the standards for inclusion as well as standards of exclusion.

The study cases were divided into two groups.

[1] Group 1: NSC (number of patients = 478, number of implants = 820)

[2] Group 2: FDP (number of patients = 456, number of implants= 790)

The database contains details about the implant, including its year of manufacture, type of anchorage, position, measurements, manufacturer and the moment of insertion and loading. Mesial and distal bone height, periapical radiolucency, mobility of implant, PI and elimination of implants were among the biological problems. Complications related to prosthetics included ceramic chipping, screw fracture, loosening and breaking of screws, implant crown decementation and prosthesis remaking. In this study, an implant and prosthesis in situ were considered to be "in function," independent of the existence or lack of biological or technological problems. Using Digora software, crestal bone height was measured on standardized peri-apical digital radiographs. All radiographs were acquired using the long cone parallel approach using the Rinn ORA positioner in order to minimize measurement error. A gap of 200 millimetres (8 inches) between the source and the skin was kept constant. HD intraoral digital sensor installed in a sensor positioner that is universal. It was requested of the patient to bite into the bite block. One examiner (EDG), who did not participate in the patient's care, estimated the bone height at bot middle as well as distal aspect from the interface of implant with abutment up to the most apical point of contact between bone and implant. The mesial as well as distal elements of every implant were determined when the prosthesis was placed; the first radiograph was obtained as a baseline assessment. The subsequent radiograph was obtained at least a year after the fitting of prosthesis but no later than eighteen months after the installation. Bone height was measured twice, minimum two weeks apart, on 10 randomly chosen baseline radiographs with the objective to assess the measurement's reliability and compute an Intra-examiner Correlation Co-efficient (ICC). All patients who were asked to come in for a yearly follow-up were provided with standard advice on oral hygiene as well as implant hygiene maintenance, including interdental brushing and flossing.

## Statistical analysis:

The SPSS program, version 24, was used to conduct the statistical analysis (IBN, USA). After accounting for the grouping of implants within patients, mixed regression models were used to evaluate the relationships between outcomes and patient and implant level characteristics. For crestal bone loss, mixed logistic regression models were employed. Each element was put into a univariate model and all patient including implant level variables were included in multivariable models.

## Results:

An ICC of 0.84 was observed that is regarded as satisfactory. In this study total 934 patients with total 1610 implants were included. Distribution of study participants according to gender, age groups, case type, medical comorbidity like diabetes mellitus, hypertension etc, smoking and implant abutment connection has been shown in Table 1. 478 (51.17%) patients included in NSC category while 456 (48.83%) patients were included in FDP category. 820 (50.93%) implants were evaluated in NSC category while 790 (49.07%) implants were evaluated in FDP category. Patients were divided in three age groups of 20-40 years age group, 41 to 60 years age group and more than 60 years age group (Table 1).

The mean distal and mesial crestal bone loss is displayed in Table 2,Table 3 and Table 4together with the outcomes of both unadjusted as well as adjusted regression analyses, based on a number of study variables like gender, age, case type, medical comorbidity, history of smoking and implant abutment connection (Table 3). On radiographs taken at baseline, during the prosthesis placement and a year later, the height of the bones was assessed. The crestal boneloss at mesial aspect in NSC cases and FDP cases was 0.31±0.44 mm and 1.24 ±0.74 mm respectively. The crestal bone loss at distal aspect in NSC case type and FDP case type was 0.37±0.55 mm and 1.30 ±0.72 mm respectively. Adjusted analyses as well non adjusted analysis of all factors for crestal bone loss at both distal as well as mesial aspect found statistically significant increased crestal bone loss in FDP patients as compared to NSC patients (p=0.001) (Table 2). Adjusted analysis of all variables for bone loss by gender, there was no statistically significant variations between male and females, however unadjusted analysis showed greater crestal bone loss at distal aspect in males as compared to females (p=0.042). There was increase in mean crestal bone loss with age at both distal as well as mesial aspect; however adjusted analysis revealed no statistically significant difference (Table 1). It was observed that adjusted analysis of all factors for crestal bone loss at both distal as well as mesial aspect found no statistically significant difference in crestal bone loss in patients with comorbidity. However, unadjusted analysis revealed greater bone loss at mesial and distal aspect in patients with medical comorbidity (Table 2). Adjusted analyses as well non adjusted analysis of all factors for crestal bone loss at both distal as well as mesial aspect found no statistically significant difference in crestal bone loss in patients with history of smoking as compared to patients with no history of smoking. It was also noticed that there was no statistically significant difference in crestal bone loss between screw retained abutment and cement retained abutment after adjusted as well as non-adjusted analysis for all variables of bone loss (Table 4). 730 (89%) of implant sin NSC case type were reported with no complications. While 608 (77%) implants of FDP case type report no complications. It was observed that cases with complications were greater in implants in FDP case type. Implant mobility was observed in 3(0.3%) implants of NSC while it was observed in 4 (0.5%) implants of FDP. PI was observed in 4 (0.4%) implants of NSC while it was observed in 5 (0.6%) implants of FDP. Loosening of screw was observed in 74 (9.0) implants of NSC while it was observed in 111 (14.0%) implants of FDP. Fracture of screw was observed in 5 (0.5%) implants of NSC while it was observed in 7 (0.9%) implants of FDP. Decementation of crowns was observed in 17 (2.1%) implants of NSC while it was observed in 26 (3.3%) implants of FDP (Table 5). 805 (98.2%) implants were in function in NSC cases, while 743 (94.0%) implants were in function in FDP cases. 5 (0.5%) implants were removed in NSC cases while 9 (1.1%) implants were removed in FDP cases (Table 6).

## Discussion:

This study was conducted to evaluate the clinical outcomes and complications of NSC and FDP in the rehabilitation of three-unit edentulous regions in the posterior jaw. The physiological issues encountered in this study were classified as mobility of implant, removal of implant and crestal bone loss. The bone loss was assessed at distal as well as mesial aspect 12 months following placement of prosthesis. Complications with the prosthesis included de-cementation of crowns, fracture of screw, loosening of screw. Gender, age groups, case type, medical comorbidity and implant abutment connection were among the variables having an impact on marginal bone being evaluated in this study. According to earlier reports, bone loss ranged 1.5-2 mm afterwards a year of function and 0.2 mm year following that [11]. In this investigation, following a year of operation, the crestal bone loss at mesial aspect in NSC cases and FDP cases was 0.31±0.44 mm and 1.24 ±0.74 mm respectively. The crestal bone loss at distal aspect in NSC case type and FDP case type was 0.37±0.55 mm and 1.30±0.72 mm respectively. Following twelve months, the implant based prostheses showed noticeably higher loss of bone, even though loss of bone in both cases of NSC and FDP was within the range documented by another study [11]. This contradicts the findings of Ravida et al. who reported that FDP had a 72 percent decreased likelihood of suffering from peri-implantitis when compared to NSC [4]. Additionally, the survival rate of FDP was superior to that of both NSC and three single implant splinted crowns. The yearly failure prevalence for three unit implant based FDPs (2.6 percent) was lower than that of three unit tooth based FPDs (3.6 percent), according to a study [4]. There was higher reduction in bone in 3-unit implant-supported FDPs after a year in comparison to NSCs in this study. It makes sense because plaque management is more challenging beneath bridge pontic. According to the results shown here, mesial as well as distal marginal bone reduction in FDPs is much higher than NSCs. Interproximal hygiene surrounding implant based fixed prosthesis is more challenging; the explanations behind this are not entirely known [1, 12]. Our findings are reasonable and suggest that NSCs are predicted to have higher survival, particularly in short-term outcomes, compared to FDPs. This is because; less bone is destroyed in the former. However, survival data is not reported here. Despite the fact that males were losing more bone at distal aspect than females, the adjusted model did not find this to be significant. Males bite strength being higher than female bite strength may be a reason for this observation [13, 14]. Furthermore, it has been demonstrated that crestal loss of bone is influenced by age and that preserving dental health gets more challenging when grip strength and vision deteriorate. The male participants in the present research were also noticeably older as compared to the female participants. The medical histories were obtained from the hospital documents and 307 patients with hypertension, diabetes or cardiovascular illness were included in the present investigation. Neoplastic as well as metabolic bone diseases did not affect any of the patients. Implant implantation in medically challenged patients is contraindicated in certain situations, but not in all cases. Almost all medically impaired patients can benefit from implants as a therapy option, although they do require exceptional plaque control, ongoing care and follow-up [15]. In cases where diabetes is inadequately managed, there is a reciprocal connection between the periodontal disease and diabetes. It may elevate the likelihood of PI and interfere with osseointegration. However, when diabetes is well regulated, the survival rate of implants is comparable to that of healthy patients. This is reported for the first six years after implant placement, but it decreases after 20 years [18]. After adjusting for every variable, the current study found that, as compared to healthy persons, medical issues did not significantly increase crestal bone loss. This is in favor of implant provision for people with poor health. After examining a variety of medical issues, a study came to the conclusion that the level of illness control may matter significantly more than the specifics of the systemic illness [19, 20-21]. This validates the earlier discovery that the loss of bone during the first year was not predicted by gender, age, ASA categorization [20]. The drugs history of patient population was not examined in this study. Further studies should examine the relationship between pharmaceuticals and implant results. It may be due to fact that non-steroidal anti-inflammatory drugs, glucocorticoids and chemotherapy, can have a negative impact on the healing of wounds [21]. After four years, smokers who smoke more than fifteen cigarettes a day have been found to have significant loss of bone [22]. In contrast, a 5-year follow-up study found no evidence of a detrimental effect of smoking upon crestal loss of bone. The effect of smoking on implant survival is still controversial but it is not a contraindication for implant therapy. The prevalence of smoking in the Middle East has increased, especially in young adults [23,24,- 26]. In the present study, there was no significant difference on crestal bone between smokers and non-smokers. This can be explained by the low number of smokers although under-reporting for cultural reasons, especially among females has been recognised [26]. A study reported that screw retained prostheses had greater crestal bone loss than cement retained. It was also found in the present study but the difference was not significant. It was probably because of the low number of cement retained implants at just 66 [16,17, 18-19]. In current research, 805 (98.2%) implants were in function in NSC cases. While 743 (94.0%) implants were in function in FDP cases. 5 (0.5%) implants were removed in NSC cases while 9 (1.1%) implants were removed in FDP cases. Consequently, there was high survivorship for both NSCs and FDPs. This is in line with the findings of a study [21], who calculated that implants bearing fixed prostheses had an overall rate of survival of 95.6 percent after five years and 93.1 percent after ten. Some assessed the survival percentage after ten years of service in FDPs be 86.7 percent [1], which is less than that stated in the current investigation. Others calculated the rate of survival following five years of functioning for NSCs to be 94.5 percent [30, 31, 32-33].

Screw loosening was the most frequent complication (12.6%) in this study followed by crown de-cementation (3.8%). A study [21] reported that veneering material fracture was the most frequent complication (13.5%) followed by 5.3% screw loosening. Because physical properties of veneering ceramic have evolved and improved, chipping is less of a problem nowadays. The study found no statistically significant differences in implant problems based on factors such as gender, age group, case type, medical comorbidity and smoking. Despite the small number of cemented cases (107), implant complications occurred less frequently in SRP than CRP. This is consistent with earlier research showing that SRP had greater technical complications while CRP had greater biological complications [23]. While CRP offers ideal occlusal design, improved aesthetics and passive alignment of the restoration, SRP necessitate precise surgical skill and prosthetic layout [23, 24-25]. There is no statistically noteworthy variation in prosthetic morbidity between NSC and FDP, according to a comprehensive systematic review [25]. According to other research, chipped ceramic prostheses accounted for the majority of prosthetic problems in FDP [9, 10, 11, 12, 13, 14, 15, 16, 17, 18, 19, 20, 21, 22, 23, 24, 25, 26]. The most common prosthetic problems with NSC were slipping of the screws, tearing of the ceramic and diminished retention [9, 10, 11, 12, 13, 14, 15, 16, 17, 18, 19, 20, 21, 22, 23, 24, 25, 26]. The most common technical problems with FDPS are veneering material breaks, slipping of the abutment or screws and reduction of retention [22]. According to the current study, problems occurred in twenty three percent of FDPs, which is considerably more than the eleven percent NSC. Additionally, consistent with other research, screw slippage was the most frequent prosthetic problem in the current study for both NSC and FDP. Greater crown-to-implant length ratios, greater inter-arch distances, insufficient tightening torque and stress creation modifies screw structure. These factors ultimately results in metal fatigue. These factors have been linked to elevated screw slippage [25, 26-27]. The primary constraints of the current investigation stem from its retrospective methodology and dependence on the precision of medical data. Besides, marginal crestal bone loss surrounding implants occurs in three dimensions. While radiographic evaluation only permits assessment of the distal and mesial surface not the buccal and lingual. Cost factor and implant manufacturer were not taken into consideration.

## Advancement to knowledge:

There was a dearth of literature comparing the use of two or three implants in the rehabilitation of three-unit edentulous regions in the posterior part of mandible as well as maxilla. This study evaluates the clinical outcomes and complications of NSC and FDP in the rehabilitation of three-unit edentulous regions in posterior jaw. It will add knowledge to clinicians in choosing the best treatment options.

## Conclusion:

The crestal bone loss was lesser in three implants with non-splinted crowns than two implants supporting a three unit bridge with an intervening pontic. The overall complications were also greater in two implants supporting a three unit bridge as compared to three implants with non-splinted crowns.

## Figures and Tables

**Table 1:** Distribution of study participants according to gender, age, case type, medical comorbidity, smoking and implant abutment connection

**Total patients= 934 Total implants= 1610**		
	No of patients n (%)	No of implants n (%)
Gender		
Male	312 (33.49)	504 (31.30)
Female	622 (66.51)	1106 (68.70)
**Age**		
20-40 years	214 (22.91)	347 (21.55)
41-60 years	412 (44.11)	760(47.20)
> 60 years	308 (32.97)	503(31.25)
**Case type**		
NSC	478 (51.17)	820 (50.93)
FDP	456 (48.83)	790 (49.07)
**Medical comorbidity**		
No	627 (67.13)	1123 (69.75)
Yes	307 (32.87)	487 (30.25)
**Smoking**		
No	812 (86.93)	1234 (76.64)
Yes	122 (13.07)	376 (23.36)
Implant abutment connection		
Screw retained prosthesis (SRP)	-	1503 (93.35)
Cement retained prosthesis (CRP)	-	107 (6.65)

**Table 2:** Mean crestal bone loss (mesially) and mean crestal bone loss (distally) one year after placement of implants by study variables like gender and age

	**Gender**		**Age (years)**		
	Male	Female	20-40	41-60	>60
Mean crestal bone loss mesially + SD mm	0.79±0.84	0.73±0.77	0.75±0.94	0.70±0.86	0.92±0.92
Unadjusted β (95% CI)	Ref	- 0.07(- 0.18, 0.05)	Ref	0.07(- 0.07, 0.19)	0.27 (0.14, 0.40)
		p= 0.244			p < 0.001
Adjusted β (95% CI)	Ref	0.01 (- 0.08, 0.10)	Ref	-0.11(-0.18,0.01)	-0.05(-0.16,0.08)
		p = 1.074			p = 0.115
Mean crestal bone loss distally (SD) mm	0.94±0.86	0.83±0.76	0.75±0.86	0.81±0.78	1.0±0.76
Unadjusted β (95% CI)	Ref	-0.12(-0.24,-0.01)	Ref	0.09(- 0.07, 0.22)	0.27 (0.19, 0.41)
		p = 0.042			p < 0.001
Adjusted β (95% CI)c	Ref	- 0.09 (- 0.19, 0.02)	Ref	-0.11(-0.21,0.02)	-0.10(-0.22,0.04)
		p = 0.105			p = 0.179

**Table 3:** Mean crestal bone loss (mesially) and mean crestal bone loss (distally) one year after placement by study variables like case type and medical comorbidity

	**Case type**		**Medical comorbidity**	
	NSC	FDP	No	Yes
Mean crestal bone loss mesially + SD mm	0.31±0.44	1.24 ±0.74	0.68±0.62	0.87±0.72
Unadjusted β (95% CI)	Ref	0.87 (0.81, 0.93)	Ref	0.18 (0.08, 0.28)
		p = 0.001		p = 0.001
Adjusted β (95% CI)	Ref	0.87 (0.82, 0.93)	Ref	0.08 (- 0.02, 0.16)
		p = 0.001		p = 0.080
Mean crestal bone loss distally (SD) mm	0.37±0.55	1.30±0.72	0.79±0.78	0.99±0.81
Unadjusted β (95% CI)	Ref	0.94 (0.88, 1.21)	Ref	0.19 (0.08, 0.30)
		p < 0.001		p = 0.002
Adjusted β (95% CI)	Ref	0.95 (0.88, 1.12)	Ref	0.09 (- 0.02, 0.18)
		p < 0.001		p = 0.071

**Table 4:** Mean crestal bone loss (mesially) and mean crestal bone loss (distally) one year after placement of implants by study variables like smoking and implant abutment connection

	**Smoking**		**Implant abutment connection**	
	No	Yes	Screw	Cement
Mean crestal bone loss mesially (SD) mm	0.68 ±0.67	0.87 ± 0.72	0.76±0.69	0.57±0.57
Unadjusted β (95% CI)	Ref	-0.02(-0.17, 0.15)	Ref	- 0.15(- 0.34, 0.05)
		p = 0.916		p = 0.401
Adjusted β (95% CI)	Ref	0.01(- 0.14, 0.13)	Ref	- 0.07 (- 0.32, 0.09)
		p = 1.246		p = 0.405
Mean crestal bone loss distally (SD) mm	0.88±0.79	0.79±0.88	0.87±0.81	0.78 ± 0.57
Unadjusted β (95% CI)	Ref	- 0.04(- 0.20, 0.16)	Ref	- 0.04 (- 0.26, 0.20)
		p = 0.839		p = 0.872
Adjusted β (95% CI)	Ref	- 0.09(- 0.23, 0.07)	Ref	0.06(- 0.24, 0.14)
p value		p = 0.310		p = 0.553

**Table 5:** Complications of implants in NSC and FDPs

	**No complications n (%)**	**Implant mobility n (%)**	**Peri-implantitis n (%)**	**Screw loosening n (%)**	**Screw fracture n (%)**	**Crown de-cementation n (%)**
NSC (820)	730 (89)	3 (0.3)	4 (0.4)	74 (9.0)	5 (0.5)	17 (2.1)
FDP group (790)	608 (77)	4 (0.5)	5 (0.6)	111 (14.0)	7 (0.9)	26 (3.3)

**Table 6:** Final outcomes of implants and prosthesis in NSC and FDP

	**In function n (%)**	**Remake prostheses n (%)**	**Removal of implant n (%)**
NSC(820)	805 (98.2)	20 (2.4)	5 (0.5)
FDP (790)	743 (94.0)	30 (3.8)	9 (1.1)
